# Carcinogenic risk of food additive AF-2 banned in Japan: a case study on reassessment of genotoxicity

**DOI:** 10.1186/s41021-023-00292-3

**Published:** 2023-12-05

**Authors:** Masami Yamada, Takayoshi Suzuki, Arihiro Kohara, Masamitsu Honma

**Affiliations:** 1 Department of Applied Chemistry, National Defense Academy, 1-10-20, Hashirimizu, Yokosuka, Kanagawa 239-8686 Japan; 2 Division of Genetics and Mutagenesis, National Institute of Health Sciences, 3-25-26, Tonomachi, Kawasaki-ku, Kawasaki city, Kanagawa 210-9501 Japan; 3 JCRB Cell Bank, National Institute of Biomedical Innovation, 7-6-8, Saito-asagi, Osaka, Ibaraki 567-0085 Japan; 4 Division of General Affairs, National Institute of Health Sciences, 3-25-26, Tonomachi, Kawasaki-ku, Kawasaki city, Kanagawa 210-9501 Japan

**Keywords:** Food additive, AF-2, Genotoxicity, Re-assessment, VSD

## Abstract

**Background:**

Carcinogenic risk assessment studies have been repeatedly improved and are still being debated to find a goal. Evaluation might be changed if new approaches would be applied to some chemicals which means that new approaches may change the final assessment. In this paper, the risk assessment of a chemical, in particular the proper carcinogenicity, is examined using the long-banned food additive, 2-(2-furyl)-3-(5-nitro-2-furyl)-acrylamide, AF-2, as a case study.

**Results:**

First, Ames tests were carried out using strains TA1535, TA100, TA1538, and TA98 and their nitroreductase-deficient strains YG7127, YG7128, YG7129, and YG7130. The results showed that mutagenic activity was reduced by about 50% in the nitroreductase-deficient strains, indicating that part of the mutagenic activity shown in Ames test was due to bacterial metabolism. Second, in vivo genotoxicity tests were conducted, including the one that had not been developed in 1970’s. Both a micronucleus test and a gene mutation assay using transgenic mice were negative. Third, assuming it is a genotoxic carcinogen, the virtual safety dose of 550 μg/day was calculated from the TD_50_ in rats with a probability of 10^−5^.

**Conclusion:**

AF-2 has been shown to be carcinogenic to rodents and has previously been indicated to be genotoxic in vitro. However, the present in vivo genotoxicity study, it was negative in the forestomach, a target organ for cancer, particularly in the gene mutation assay in transgenic mice. Considering the daily intake of AF-2 in the 1970s and its virtually safety dose, the carcinogenic risk of AF-2 could be considered acceptable.

**Supplementary Information:**

The online version contains supplementary material available at 10.1186/s41021-023-00292-3.

## Background

Food safety is a major concern for the public these days, especially the safety of chemical substances contained in daily foods, such as food additives. In particular, when a compound is found to be carcinogenic, proper evaluation is an important issue. Theoretically, a linear dose-response model with no threshold has been applied when assessing the risk of genotoxic compounds to human health [1, 2]. However, now that the mechanism of carcinogenesis is more precisely understood [3], non-genotoxic carcinogens that do not directly damage DNA are considered to have a toxicity threshold [4, 5]. This means that the risk of cells becoming cancerous due to exposure to a non-genotoxic compound may be practically negligible if the exposure level of that compound is lower than a certain threshold. Therefore, the determination of genotoxic hazard is important in determining the direction of risk management for carcinogens. However, the sensitivity of genotoxicity tests is high, and the fact is that their positive responses are not always closely related to high human carcinogenicity [6]. In such cases, the weight of evidence and mechanism of action, MOA, are important considerations when making decisions about the safety assessment of a chemical. The latter information is particularly important because it leads to the establishment of exposure thresholds when assessing the health risk of a chemical.

The opportunity for humans to ingest carcinogens in daily life is through a variety of sources, including food, drinking water, and air. Under these circumstances, it is rather unbalanced and inefficient to stick to a specific chemical and discuss its carcinogenicity or genotoxicity regardless of the exposure level. Therefore, it is reasonable to accept some level of risk when the exposure or intake of a chemical is sufficiently small. From this perspective, to evaluate the genotoxicity and carcinogenicity of trace amounts of food additives and pesticide residues in food, three items are required for evaluation: hazard identification, evaluation of exposure levels, and newly developed toxicity tests that can give more accurate data for considering MOA [7–9]. Therefore, it is an interesting and important attempt to re-evaluate exposure risks using these new methods, even for chemicals that have already been evaluated.

**Fig. 1:**
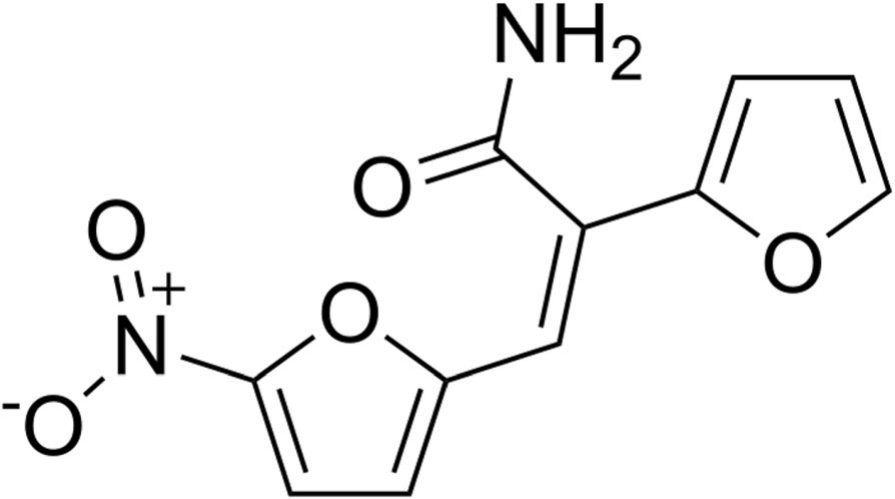
The structure of AF-2

The nitrofuranoid 2-(2-furyl)-3-(5-nitro-2-furyl)-acrylamide (AF-2, Fig. 1) is used as a positive control for TA100 and TA98 in the Ames test, an in vitro genotoxicity test. Its high induction ability is considered to be bacterial-specific, since it does not show mutagenicity in the absence of the bacterial-specific plasmid pKM101 [10]. A similar example, the case of 1-nitropyrene, supports this idea that high mutagenicity is specific to bacteria: the compound 1-nitropyrene is known to be a potent mutagen, showing very high mutagenicity at nanogram-level doses in the Ames test [11]. It is classified by the International Agency for Research on Cancer, IARC, as “probably carcinogenic to humans” group 2A and is therefore considered to be of very high risk to humans. Its high mutagenicity is reportedly caused by metabolic activation via “classical” nitroreductases, NRs, which are specific to bacteria [12–14]. This possibility is presumably also true for AF-2, which has a nitro group; AF-2 has been used in Japan since 1965 as a sanitizer for soybean curd, fish sausage, and noodles. In fact, it was an alternative to the previous preservatives nitrofurazone and nitrofuryl acrylamide because of its low chronic toxicity [15]. However, in the 1970s, chromosomal aberrations, (CAs), in human lymphocytes [16] and mutagenicity in *Escherichia coli* [17, 18] of AF-2 were reported (Table 1). Subsequently, a carcinogenicity study of AF-2 was conducted using ddY mice, which developed malignant tumors in the forestomach [19]. AF-2 was then finally banned in 1974 because of its suspected mutagenicity and carcinogenicity. The above is a brief history of AF-2 regulation some 40 years ago. However, at that time, its mutagenic mechanism and degree of carcinogenicity were not fully examined as part of the risk assessment.

**Table 1:** Results of genotoxicity tests for AF-2

Class	Assays	Cells/species	Doses	Results	References
In vitro	Rec-assay	*B. subtilis*	2 μg/plate	Positive	Kada [18]
	Reversion assay	*E. coli* H/r30R	0.1–4 μg/mL	Positive	Kondo & Ichikawa-Ryo [17]
	Reversion assay	*E. coli* WP2*hcr*^*−*^	0.5, 1.0 μg/plate	Positive	Kada [18]
	Ames test	*S. typhimurium* TA100, TA98	0.02 μg/plate	Positive	McCann et al. [10]
	Chromosomal aberration	human lymphocyte	0.5–50 μM [=0.12–12.4 μg/mL]	Positive	Tonomura & Sasaki [16]
	Unscheduled DNA synthesis	human fibroblast	80 μM [=19.8 μg/mL]	Positive	Tonomura & Sasaki [16]
	Gene conversion	Yeast	50, 100, 200 μg/mL	Positive	Murthy & Sankaranakarayan [37]
	Gene mutation	Chinese hamster, V79	50–400 μM [=12.4–99.2 μg/mL]	Positive	Wild [36]
	Gene mutation	mouse lymphoma, L5178Y Ala32	1–50 μg/mL	Positive (+S9)	Nakamura et al. [45]
In vivo	Micronucleus assay	rat; Long-Evance bone marrow	60, 120, 240 mg/kg; i.p.	weakly positive	Goodman et al. [28]
	Micronucleus assay	mouse; CD-1, MS/Ae rat; SD peripheral blood	50, 100, 200 mg/kg; i.p. 25, 50, 75, 100 mg/kg; i.p.	Weakly positive	Higashikuni et al. [39]
	Chromosomal aberration	rat; Long-Evance bone marrow	30–240 mg/kg; gavage	positive	Sugiyama [38]

Therefore, this paper uses this old banned food additive as a case study to reconsider the appropriate risk assessment, especially the carcinogenicity of the chemical, using the peripheral blood micronucleus, MN, test in rats and in vivo gene mutation in transgenic mice that can be examined for organ-specific mutations, including carcinogenic target organs assay to understand their MOA [7–9]. In addition, the Ames test was performed using strains without bacteria-specific NRs.

## Materials and methods

### Chemicals used in this study

AF-2 (CAS No. 3688-53-7, Purity 98%) and dimethylsulfoxide (DMSO, CAS No. 67–68-5) were purchased from Wako Pure Chemical Industries, Ltd. (Osaka, Japan). Dibenzo[*a,l*]pyrene (DBP, CAS No. 191–30-0) was purchased from SUPELCO (PA, USA).

### Ames test

The strains used in the Ames test were listed in Table 2: TA1535, TA100, YG7127, YG7128, TA1538, TA98, YG7131 and YG7132. They are all *Salmonella enterica* subsp. enterica serovar Typhimurium (*S. typhimurium*). YG7127, YG7128, YG7131 and YG7132 specifically lack an *nfsB* gene, which encodes an NR in TA1535, TA100, TA1538 and TA98, respectively [20]. TA100, YG7128, TA98 and YG7132 harbour pKM101, which contains the *mucAB* genes that encode PolRI, a translesion DNA polymerase [21].

**Table 2:** List of strains used in the Ames test

Strains	Characteristics	Genetic variation		Source
*S. typhimurium*		NR	pKM101	
TA1535	*hisG46* ^a)^, *gal*, Δ(*chl*, *uvrB*, *bio*) *rfa*	Wild type	No	[26]
TA100	Same as TA1535, but harbours pKM101; Ap^r b)^	Wild type	Yes	[10]
TA1538	Same as TA1535, but *hisD3052* ^c)^ instead of *hisG46*	Wild type	No	[26]
TA98	Same as TA1538, but harbours pKM101; Ap^r^	Wild type	Yes	[10]
YG7127	Same as TA1535, but an *nfsB* gene is deleted; Km^r d)^	Deleted	No	[20]
YG7128	Same as YG7127, but harbours pKM101; Ap^r^, Km^r^	Deleted	Yes	[20]
YG7131	Same as TA1538, but an *nfsB* gene is deleted; Km^r^	Deleted	No	[20]
YG7132	Same as YG7131, but harbours pKM101; Ap^r^, Km^r^	Deleted	Yes	[20]

a) *hisG46* is a base-substitution mutation, CTC to CCC, at the 69th codon of the *hisG* gene

b) Ap^r^ means ampicillin resistant

c) *hisD3052* is a frameshift mutation, CGCGCG to CGCGCGCG, in the *hisD* gene

d) Km^r^ means kanamycin resistant

The test chemical AF-2 was dissolved in DMSO and concentrations used in the tests are 0.2, 0.1, 0.05, 0.025, and 0.013 μg/plate. The S9, rat liver extract for metabolic activation, was purchased from Kikkoman Corporation (Chiba, Japan). The mutagenicity assay was carried out with pre-incubation described by DM Maron and BN Ames [22] in triplicate plates for each dose. Briefly, an overnight culture prepared by inoculating nutrient broth (5 mL) with frozen cells was subjected to the assay. A mixture containing 0.1 mL of the overnight culture, 0.1 mL of AF-2 solution and 0.5 mL of S9 mix was incubated for 20 min at 37 °C. When metabolic activation was not required, 0.5 mL of 1/15 M phosphate buffer, pH 7.4, was added in place of the S9 mix. After the pre-incubation, the mixture was poured onto agar plates with 2 mL of soft agar and incubated for 2 days at 37 °C. The number of revertants per plate was counted.

### Animals, diet and housing conditions

Male 6-week-old Muta™Mice, λgt10*lacZ-*introduced CD2F_1_ (BALB/C x DBA2), were supplied by Covance Research Products (PA, USA) for use in the transgenic mouse gene mutation assay (Tg assay), and the MN assay. All animals were housed in polycarbonate cages at four per cage under specific pathogen-free, standard laboratory conditions: room temperature 23 ± 2 °C and relative humidity 60% ± 5%. The animals experienced a 12:12-h light - dark cycle and had free access to CRF-1 basal diet (Oriental Yeast Company, Tokyo, Japan) and tap water.

### Treatments of animals

A 6-week-old male Muta™Mice (ca. 25 g body weight) were acclimatized for 1 week before use and divided into three groups, each of which consists of four mice. Based on its LD_50_ in mice [23], 120 mg/kg, 25% of the LD_50_, of AF-2 was administered intragastrically at a concentration of 1 mg/mL once a week, four times. The positive control, 6 mg/kg DBP and the vehicle, olive oil, were intraperioneally administrated to the positive and negative control groups, respectively, at the same time as AF-2 was administered to the test group. The protocol for this study was approved by the Animal Care and Utilization Committee of the National Institute of Health Sciences.

### Peripheral blood MN assay

48 hours after the first and the second administration of AF-2, peripheral blood (5 μl) was collected without anticoagulant from the tail blood vessel, placed on an acridine orange-coated glass microscope slide, covered with a cover slip and supravitally stained [24]. 1000 reticulocytes, RETs, per animal were analysed by fluorescence microscopy within a few days of slide preparation, and the number of cells with micronuclei was recorded.

### A transgenic mice gene mutation assay

Animals were killed 7 days after the final treatment by cervical dislocation. Colon, forestomach, liver and spleen, which were reported as target organs for carcinogenesis in rodents, were collected, quickly frozen in liquid nitrogen and then stored in a deep freezer at − 80 °C until analyses could be performed. The isolation of genomic DNA from the tissue samples was carried out as indicated by the manufacturer’s protocol (Covance Manual, 1996). Briefly, homogenised tissues were incubated with ribonuclease and proteinase K, and impurities, mainly proteins, were removed using a phenol-chloroform mixture and chloroform. The DNA was precipitated with ethanol and dissolved in TE-4 buffer (10 mM Tris· HCl pH 8.0 containing 4 mM ethylene diamine tetra acetic acid).

The *lacZ* transgene, integrated into the lambda phage vector (λgt10), was recovered using in vitro packaging reactions. The DNA solution (10 μL), adjusted to a concentration of 0.5–1.5 mg DNA/mL, was gently mixed with 10 μL of Transpack® (Stratagene, La Jolla, CA, USA) and incubated at 37 °C for 3 hours. The volume of the mixture was increased to 1 mL with SM buffer (NaCl, 5.8 g; MgSO_4_-7H_2_O, 2 g; 1 M Tris-Cl (pH 7.5) buffer, 50 mL; 2% gelatine soln., 5 mL; per liter). The *lacZ* mutant frequency, MF, was determined by positive selection with *galE*^−^ of *E. coli*, according to the manufacturer’s manual (Corning Hazleton, 1996). In this experiment, diluted LB medium, which consists of 0.25% Tryptone, 0.125% yeast extract and 0.765% NaCl adjusted to pH 7.0, was used for the bottom agar (1.5%, 10 mL per plate) and the top agar (0.7%). Briefly, the packaged phage (500 μL) was added to 2 mL of *E. coli* C (*lac galE*) culture and incubated at room temperature for 20 min to allow adsorption of the phage particles to the bacteria. For titration, 1 mL of the phage-bacteria solution prepared above was mixed with 23 mL of top agar containing 10 mM MgSO_4_. The mixture was plated over four plates (9 cm) containing 6 mL of bottom agar, 6 mL for each. The remaining phage-bacteria solution was mixed with 21.5 mL of top agar containing P-gal (3 mg/mL) and poured onto four plates. The plates were incubated at 37 °C overnight.

Selection for *cII* mutants was carried out according to the method of Jakubczac et al. [25] with slight modifications. Briefly, the packaged phage was added to 1-mL culture of *E. coli* G1225 (Δ(*mcrA*)*183* Δ(*mcrCB-hsdSMR-mrr*)*173 endAl supE44 thi-1 gyrA96 relA1 lac*^c^ *supF hflA*::Tn*5 hflB29*::Tn*10*) and incubated at room temperature for 20 min. For the titration, appropriately diluted phage solution was mixed with 200 μL of culture for *E. coli* G1225. The phage-bacteria solution was mixed with 14 mL (for selection) or 6 mL (for titration) of LB top agar containing 10 mM MgSO_4_ and plated onto five or two plates, respectively. The plates were incubated at 25 °C for the positive selection of *cII* mutants or at 37 °C for the titer of total phages for 48 h.

A wild-type phage recovered from the Muta™Mouse has a *cI*^−^ phenotype, which permits plaque formation with an *hfl*^−^ strain like G1225 at 37 °C but not at 25 °C. The MF was calculated as follows: MF = total plaques on selection plates/(total plaques on titer plates × dilution factor).

### Statistical analysis

The difference in MF between control and treated groups in both in vivo experiments was evaluated with a one-side test using the Poisson regression with quasi-likelihood. Statistical significance was defined as *P* < 0.05.

## Results

### Ames test

To confirm that pKM101 is required to induce mutagenicity of AF-2, the bacterial reverse mutation assay was carried out using TA1535 and TA1538 comparing their pKM101-harbouring strains, TA100 and TA98, without a metabolic activation system. The AF-2 did not exhibit its mutagenicity without pKM101 (data not shown). There is no differences when the strains lack a nitroreductase, in YG7127 and YG7131, an NR-deficient derivative of TA1535 and TA1538, respectively (data not shown).

**Fig. 2:**
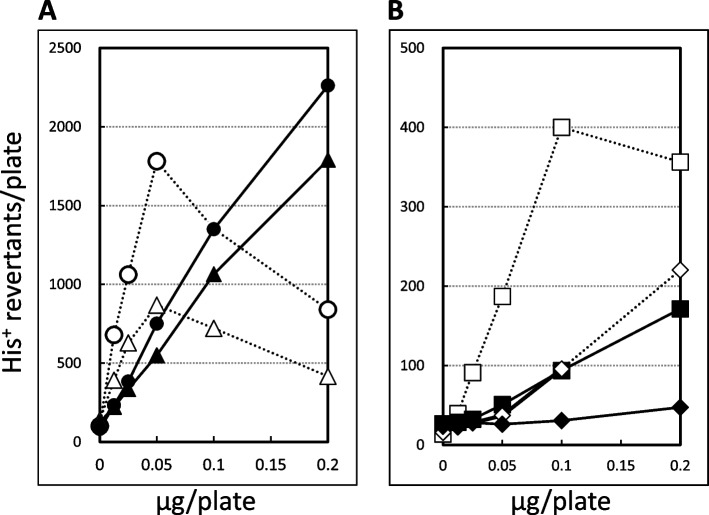
Mutagenicity of AF-2 in the Ames test and the influence of bacterial nitroreductase on its mutagenicity (**A**) Base substitutions and (**B**) Frameshift mutations ○, TA100 without S9 mix; ●, TA100 with S9 mix; △, YG7128 without S9 mix; ▲, YG7128 with S9 mix; □, TA98 without S9 mix; ■, TA98 with S9 mix; ◇, YG7132 without S9 mix and ◆, YG7132 with S9 mix. The concentrations used in the tests were 0, 0.0125, 0.025, 0.05, 0.1 and 0.2 μg/plate as indicated at the X-axes. Each plot indicates a mean value for the number of revertants on triplicate plates

Next, the Ames test was conducted in strains proficient and deficient in a nitroreductase, i.e., TA100 and TA98 and their nitroreductase deficient counterparts, YG7128 and YG7132. The assay was carried out both with and without S9mix to determine the effect of exogenous metabolic activation, S9 mix. Figure 2A shows that base substitution was significantly decreased when the strain lacked an NR, especially without S9mix. As shown in Fig. 2B, YG7132, an NR-deficient derivative of TA98, showed one-third of the number of revertants shown by the parent strain TA98 regardless of exogenous metabolic activation. In addition, S9 mix decreased the mutagenicity of AF-2 in any strains.

### Peripheral blood MN assay

MN test data in the Muta™Mouse did not indicate statistically significant effects on MN frequency (Table 3) 48 hours after the first and the second administration i.g. treatment with AF-2 (120 mg/kg) compared to the vehicle control group. The results were indicated in the mean incidence of micronucleated RETs (MNRETs) per 1000 RETs. DBP (6 mg/kg bw), a positive control, induced a substantially significant effect on MN frequency.

**Table 3:** Induced micronuclei in vivo

Treatment	Dose	MNRETs per 1000RETs				Mean ± SD (%)
First 48 h						
Olive oil	10 mL/kg	0	3	1	2	0.15 ± 0.11
AF-2	120 mg/kg	1	3	2	5	0.28 ± 0.15
DBP (positive control)	6 mg/kg	11	2	3	11	0.68 ± 0.43
Second 48 h						
Olive oil	10 mL/kg	1	3	0	3	0.18 ± 0.13
AF-2	120 mg/kg	0	1	2	1	0.10 ± 0.07
DBP (positive control)	6 mg/kg	8	68	53	52	4.53 ± 2.24

### A gene mutation assay using transgenic mice

To investigate the mutagenic effects of AF-2 in vivo, Muta™Mice were treated with AF-2, and gene mutation assays were carried out. Mutations are to be generated in the transgenes *cII* and *lacZ* on the lambda phage vector integrated in the genome of the mice. No significant increases of mutation frequencies were detected between the AF-2 treated group and the vehicle control group at each organ, colon, forestomach, liver and spleen, which was collected from Muta™Mice (Table 4, Supplementary data). In this paper, the Tg assay was not conducted on the mammary gland, which is a carcinogenic target organ of AF-2 in rats, because it is not a target in mice and the mammary gland is too small to prepare samples for the Tg assay in mice. Treatment of the transgenic mice with DBP, a positive control, resulted in an increase in MF in all the organs investigated; the results were especially significant in the spleen.

**Table 4:** Mutant frequencies in the transgenic mouse gene mutation test

Target genes	Chemicals	Doses	Mutant Frequency (×10^−6^)			
			Spleen	Liver	Colon	Forestomach
*lacZ*	Olive oil	10 mL/kg	40.4 ± 24.5	62.9 ± 10.1	82.5 ± 76.6	30.8 ± 2.7
	AF-2	120 mg/kg	30.4 ± 4.3	66.1 ± 8.1	39.9 ± 5.4	57.0 ± 30.7
	DBP	6 mg/kg	535.5 ± 230.0	280.7 ± 24.6	189.7 ± 40.1	220.4 ± 47.8
*cII*	Olive oil	10 mL/kg	18.1 ± 12.5	31.1 ± 14.6	77.4 ± 65.8	42.6 ± 32.0
	AF-2	120 mg/kg	39.7 ± 22.9	45.9 ± 32.0	31.6 ± 10.6	29.8 ± 12.9
	DBP	6 mg/kg	348.9 ± 175.0	77.3 ± 28.4	123.7 ± 37.9	99.7 ± 15.4

Administration was carried out intraperitoneally for olive oil and DBP and orally for AF-2

See Supplementary Data for detailed figures on which the calculations are based

## Discussion

The use of AF-2 as an additive for the preservation of food was approved in the 1960s in Japan. CAs resulting from treatment of human cells with AF-2 were first described in the 1970s, [16], but the newly developed bacterial reverse mutation assay, the Ames test, could not detect its mutagenicity at that point because the strains used in the study, TA1535 and TA1538, were insensitive to the AF-2 [10]. This fact prompted researchers to develop TA100 and TA98, both of which have become official standard strains in the Ames test; AF-2 has been a positive control for the Ames test since then [10, 26, 27]. Positive responses to AF-2 were subsequently reported in several in vivo genotoxicity tests, including the MN assay and the CAs test [28]. The results from carcinogenicity studies are summarised in Table 5. No adverse effects were observed in chronic toxicity tests in rats or mice fed food containing 0.2% AF-2 for 24 months [29]. However, the carcinogenicity was exhibited by AF-2 in some reports [30–33]. Ochiai et al. carried out a carcinogenicity test using mice-fed diets containing 0, 0.05, 0.15%, or 0.45% AF-2 for 18 months. They found malignant tumors, including squamous cell carcinoma, developed in a dose-dependent manner in the forestomach [19]. Finally, AF-2 was removed from the list of designated food additives in Japan in September 1974. This case illuminated the importance of genotoxicity tests; they have been incorporated into safety guidelines for medicines and agrochemicals, as well as for food additives. Unfortunately, however, genotoxicity tests at that time were not sufficiently validated, and the risk assessment procedure was not the same as it is at present. Hence, we re-evaluated the carcinogenic risk of AF-2 through the mechanism of its action using genotoxicity tests, which had not been developed at that time.

**Table 5:** Results of carcinogenicity tests for AF-2

Animal/species	Doses	TD_50_ mg/kg/day	malignancy-observed tissues	References
Rat, SD	0.2%, in food 46 weeks		breast cancer adenocarcinoma	Cohen et al. [30]
Rat, Wistar	0.4%, in food, 18 months	74.7	mammary, tumor forestomach, papillomas	Takayama and Kuwabara [46]
Rat, Wistar			mammary tumors	Takayama and Kuwabara [32]
Mouse, CDF_1_	0.08/0.4%, in food 18 months	714	forestomach, tumors	Takayama and Kuwabara [32]
Mouse, ICR/JCL	0.08/0.4%, in food 440 days	95.0	forestomach, carcinoma/ papillomas	Yokoro et al. [47]
Mouse, ddY	2500 mg/kg, in food 308 days	72.9	forestomach, carcinoma/ papillomas	Sano et al. [31]
Mouse, ddY	0.05/0.15/0.45%, in food, 18 months	550*	forestomach, carcinoma	Ochiai et al. [19]

*See discussion for calculation method

### [Ames test]

First, a bacterial reverse mutation assay, the Ames test, was conducted in strains proficient and deficient in a nitroreductase, i.e., TA1535, TA1538, TA98 and TA100 and their nitroreductase deficient counterparts. An increase in the number of reverted colonies was observed in pKM101 strains (TA98 and TA100) but not in TA1535 and TA1538 ones. We verified the propriety of the positive result for AF-2 in the Ames test. Our results confirmed that AF-2 shows strong mutagenicity but only in TA100 and TA98, which harbour pKM101, and not at all in their parent strains, TA1535 and TA1538 [10]. The reason for the mutagenic response in TA100 and TA98 is that the DNA mutagenesis occurs through translesion DNA synthesis, TLS, done by DNA PolRI, which is encoded by the *mucAB* genes on the bacteria specific plasmid pKM101 [21]. The TLS polymerase can bypass the lesion on DNA and induce a mutation while a replicative DNA polymerase generally stalls at the lesion on DNA [34]. There are several kinds of the TLS polymerases in bacteria as well as mammals, and specificity of bypassed adducts depends on the activity of each TLS polymerase. Thus if we consider that the nature of the TLS DNA polymerases is different in bacteria and mammals, i.e., polRI can overcome the DNA adduct of AF-2 and induce mutation, but the mammalian enzyme cannot and therefore does not induce mutation, this explains the strong mutagenicity exhibited by AF-2 in the Ames test, which might be a bacteria-specific phenomenon.

The mutagenic activity was reduced in nitroreductase deficient strains (Fig. 2A and B). The results would give us another reason that the mutagenesis observed with AF-2 is partially bacteria-specific, that is, the reduction of the nitro group by bacterial NR(s) in the test strains. Suter et al. [35] reported that AMP397, a heterocyclic nitro compound, exhibited a strong mutagenic response in TA98 and TA100 without S9 mix, but the mutagenicity was not observed in NR knockout strains, or when the compound was treated with S9 mix. Mutagenicity of this compound could not be detected by in vitro tests using mammalian cells, in the MN test, or in the mouse lymphoma assay. Nor could mutagenicity be detected in in vivo tests such as the MN assay, the Tg assay, the comet assay, or the DNA binding assay. Thus, the mutagenic activity of AMP397 was proved to be completely dependent on NR activity, and AMP397 was concluded to be specifically mutagenic to bacteria. Of course, AF-2 is a different case because its mutagenicity still remains in NR-defective strains (Fig. 2A and B). The possibility cannot be neglected that AF-2 is activated through nitroreduction by other NR activities in mammalian cells. Even so, it is a fact that the mutagenicity of AF-2 was decreased less than half in Ames test if the strain lacks the NR. Our results suggest that more precise investigation of mechanisms would be important to discuss the results of genotoxicity.

### [in vitro tests]

In addition to the Ames test, there were more reports about the mutagenicity of AF-2 using in vitro tests summarized in Table 1, and AF-2 can be concluded to have clear genotoxicity in vitro without S9. Precisely, Goodman et al. [28] reported that many of nitrofurans exhibited strong mutagenicity in TA100 and TA98, while AF-2 was reported to give a weak response in several in vitro assays, such as a gene mutation assay in V79 cells [36] and a gene conversion assay in yeast cells [37]. However, the CAs were reported in human lymphocyte [16]. We thought that results in human cells might have more important implications when considering risk assessment, and decided to examine the in vivo genotoxicity of AF-2.

### [in vivo tests]

Compared with in vitro tests, not many reports have been published concerning the genotoxicity of AF-2 in vivo (Table 1). Goodman et al. reported that MN in the bone marrow was slightly but statistically significantly induced by intraperitoneal treatment of rats with AF-2 at a dose of 240 mg/kg, the induction level was 0.49% ± 0.32% in comparison to the control, 0.13 ± 0.06% [28]. Sugiyama et al. [38] observed a dose-dependent increase in the number of CAs in bone marrow cells of rats after oral administration of 15–240 mg/kg of AF-2, but the frequency of CAs was only 4.00% at the maximum dose and1.21% at the minimum dose, which is a weak response although the difference was reported as “statistically significant”. AF-2, therefore, is clearly genotoxic in vitro while its genotoxicity in vivo remains unclear. Thus, the unclear results of the in vivo reports as well as the positive results in human cells promote us to determine the mutagenicity of AF-2 in this study using a MN assay and a Tg assay.

Higashikuni et al. reported that AF-2 is a weak in vivo clastogen because MN frequency was elevated by AF-2 markedly in MS/Ae mice and very slightly in ICR and CD-1 mice [39]. As the authors also mentioned, the interpretation of data obtained with MS/Ae mice is rather difficult because the strain shows higher sensitive to mutagens in MN test, but the involved mechanism is not clarified [40]. Their conclusion is that AF-2 is narrowly clastogenic only at lethal doses. In the present study, MN was not induced by the treatment of AF-2 in mice. No mutagenicity was detected in the liver, forestomach, colon, or spleen (Table 4). The forestomach was a target organ for carcinogenesis by AF-2 in several studies but no increase of gene mutations was observed in the Tg assay. This suggests that the genotoxicity of AF-2 is not a cause of cancer generated in the forestomach (see below). Considering these results together with the negative result in MN inducibility (Table 3), the genotoxicity of AF-2 does not appear to be a cause of tumor. The carcinogenicity of relatively high doses of chemicals in the forestomach (directly exposed organs) may be due to an artificial effect that is cytotoxicity. Therefore, it may not be a carcinogen in humans [41].

Getting the ideas in shape here, AF-2 is genotoxic in vitro, but not in vivo*,* especially in forestomach, which is the target organ of AF-2 in the carcinogenicity tests in mice. The genotoxicity of AF-2 does not appear to contribute toward its carcinogenicity, which leads to the conclusion that AF-2 is not a genotoxic carcinogen. This conclusion is opposite to that of the Japanese regulation 40 years ago. And now, the identification and evaluation of genotoxicity of food-related chemicals are still controversial in Food Safety Commission in Japan [42].

### [carcinogenic risk assessment]

Based on the above, we cannot conclude that AF-2 is mutagenic in vivo, but it is clearly mutagenic in vitro, and we will evaluate the carcinogenic risk of AF-2 according to recent procedures of risk assessment using a non-threshold model [43]. The no-threshold model is based on the idea that “even a potentially carcinogenic chemical substance has a very small risk of carcinogenesis if its concentration is sufficiently low, and can be considered practically safe if its level of risk is a socially acceptable risk level. This amount is called the “virtually safe dose (VSD)”, and the risk level is considered negligible or acceptable. In this case, a lifetime risk level of 10^−6^ to 10^−5^ for cancer is used as an acceptable risk, and the VSD is generally obtained by a multi-stage model or linear extrapolation from the TD_50_ obtained in carcinogenicity studies using rodents. In the case of linear extrapolation, the VSD is obtained by dividing the TD_50_ by 50,000.

Here, as the carcinogenicity test data, we used the chronic toxicity test (carcinogenicity test) conducted by Ochiai et al. in mice [19], which was the basis for the ban on the use of AF-2 by the then Ministry of Health and Welfare. As a result of feeding 0, 0.05, 0.15, and 0.45% of AF-2 in the diet, swelling recurrence occurred in the anterior gastric region of 0, 12.1, 44.4, and 58.8% of the animals, respectively. Based on the least-squares formula of this data, the dose that causes swelling in 50% of the mice (TD_50_) is 0.33% of the mixed diet. The average body weight of the mice and the average food intake per day are assumed to be 30 g and 5 g, respectively. From this, the average daily intake of AF-2 in mice that reaches TD_50_ is calculated to be 5 g × 0.33% = 0.0165 g and converting this to kg, 0.0165 g X (1000/30) = 0.55 g/kg (= daily intake per kg body weight at which AF-2 is carcinogenic in half of animals). Linear extrapolation to a probability of 10^−5^, i.e., the accepted lifetime risk level used, is achieved by simply dividing the TD_50_ by 50,000, 0.55 g/50,000, generating a virtual safety dose, VSD, which is approved as acceptable intake for food-related chemicals and impurities in pharmaceuticals [43]. Thus, the VSD of carcinogenic risk level of 10^−5^ of AF-2 is calculated to be 0.011 mg/kg/day and the acceptable daily intake for a human weighing 50 kg is 0.55 mg/day.

For the exposure assessment, the annual production of AF-2 was reported to be about 3500 kg in Japan in 1973 [44]. Assuming that all of this material was used in food, 5% of it, or 175 kg, was estimated to remain in food using the decay factor 0.95 [17]. Using the following numbers, the average of daily intake of AF-2 can be calculated to be 3.7 μg/day (=0.073 μg/kg weight/day); Japanese population at that time, 105 million; 365 days per year, compensation number; 0.8 [44]. The VSD calculated by assuming the lifetime cancer risk to be 10^−5^ is 0.011 mg/kg/day (see above), which means that the cancer risk of AF-2 at that time was 1/150 of VSD.

It should be noted, however, that the results of the above risk assessment are based on the report by Ochiai et al. [19]. In addition, although direct extrapolation from TD_50_ was used here for extrapolation of VSD, in recent years, multi-stage models and extrapolation from BMDL_10_ (Benchmark dose level with a 10% extra risk) have also been used, and the calculated values in such cases will be different. However, even taking these into account, the carcinogenic risk of AF-2 at that time was not considered to be very high. Incidentally, AF-2 is classified as Group 2B (possibly carcinogenic to humans) in the IARC carcinogenicity classification [23]. Nevertheless, the estimated average daily intake of AF-2 at that time was 3.7 μg/day, which is above the common TTC, threshold of toxicological concern, level of 1.5 μg/person/day. The TTC is a concept currently used for risk management of many chemicals and is based on the idea that even if a substance is a carcinogen, if the daily intake is less than 1.5 μg/person, there will be little or no substantial health hazard. Currently, the Food Safety Commission, even if this issue were to arise, would not approve the use of AF-2 unless the Tg assay proves that AF-2 is not mutagenic in all carcinogenic tissues, since it is an Ames-positive carcinogen.

## Conclusion

In conclusion, AF-2 is carcinogenic in rodents and has long been noted to be genotoxic in vitro. However, in the present in vivo genotoxicity study, the Tg assay was negative, especially in the forestomach, a target organ for cancer. Furthermore, considering the daily intake of AF-2 in the 1970s and its VSD, the carcinogenic risk of AF-2 is considered acceptable. It is also reasonable to conclude that AF-2 is not genotoxic in vivo since it did not show positive results in the Tg test even not in accordance with OECD guidelines. The review of past chemical regulations has proven to be worthwhile. It is hoped that more detailed Tg assay according to the guidelines will support this result in the future.

### Supplementary Information

**Additional file 1.**

## Data Availability

All data generated during this study are included in this published article.

## Abbreviations

*AF-2*: 2-(2-furyl)-3-(5-nitro-2-furyl)-acrylamide
*TD*_*50*_: toxic dose, 50%; median toxic dose
*MOA*: mechanism of action
*IARC*: International Agency for Research on Cancer
*NR*: nitroreductase
*MN*: micronucleus
*DMSO*: dimethyl sulfoxide
*DBP*: dibenzo[*a,l*]pyrene
*LD*_*50*_: lethal dose, 50% kill; median lethal dose
*RET*: reticulocyte
*MF*: mutant frequency
*TLS*: translesion DNA synthesis
*CAs*: chromosomal aberrations
*CPDB*: cancer prediction database
*VSD*: virtual safety dose
